# Supplementary material for: Hepatitis C Virus Down-Regulates the Expression of Ribonucleotide Reductases to Promote Its Replication
Source: Pathogens. 2023 Jun 29;12(7):892. doi: 10.3390/pathogens12070892 (PMC10383090; doi:10.3390/pathogens12070892)
Supplement: Supplementary file 1 [file pathogens-12-00892-s001.zip › pathogens-2432716-supplementary.pdf]

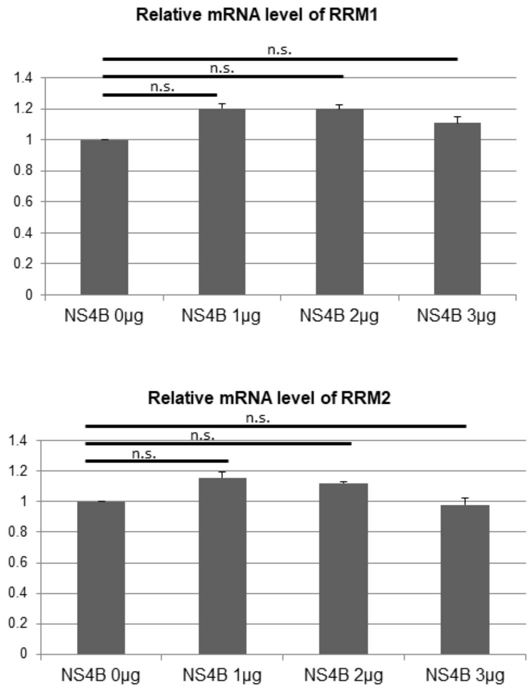

Supplementary Figure S1. Real-time RT-PCR analysis of the mRNA expression of RRM1 (Upper) or RRM2 (Bottom) 48 hrs after the transfection of various amount of vectors and/or plasmids expressing NS4B protein into Huh7 cells.

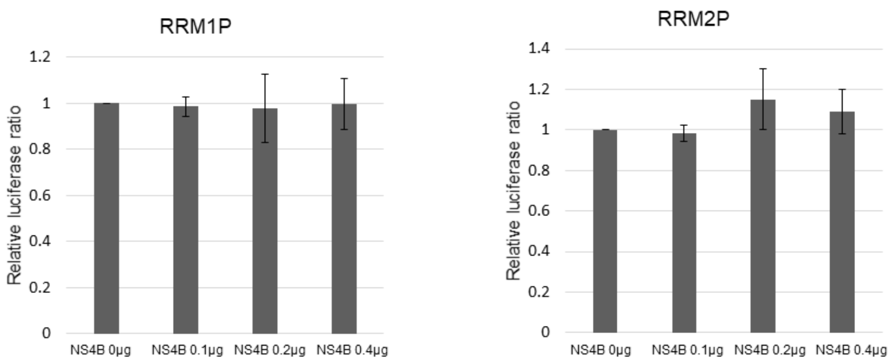

Supplementary Figure S2. Various luciferase reporter assays were performed 48 hrs after transfection of reporters for promoter activity of RRM1 (left) or RRM2 (right) and various amount of vectors and/or plasmids expressing NS4B protein into Huh7 cells.

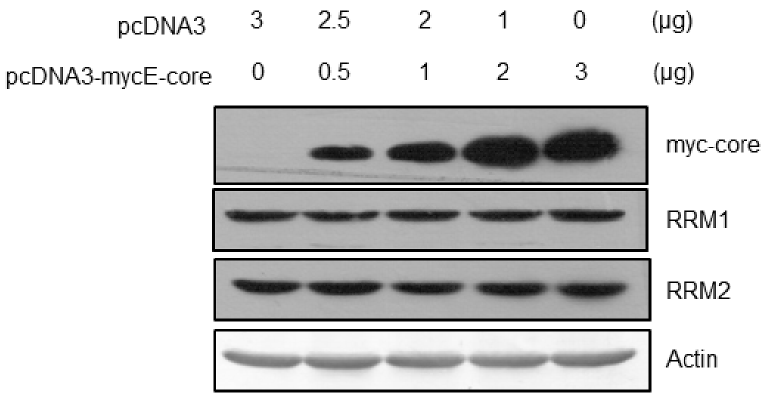

Supplementary Figure S3. Western blotting analysis of RM1 or RRM2 in Huh7 cells transfected with various plasmids as indicated. Forty-eight hrs after transfection, protein samples derived from these cells were analyzed with myc tag, RRM1 or RRM2.

(A)

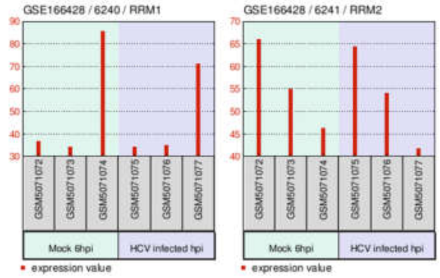

		RRM1		RRM2	
		Value	Average	Value	Average
Mock Huh-7.5 6 hpi	GSM5071072	36.97		66.08	
	GSM5071073	34.51	52.48667	55.11	55.86333
	GSM5071074	85.98		46.4	
HCV infected Huh-7.5 6 hpi	GSM5071075	34.6		64.46	
	GSM5071076	35.18	47.06667	54.22	53.54
	GSM5071077	71.42		41.94	

(B)

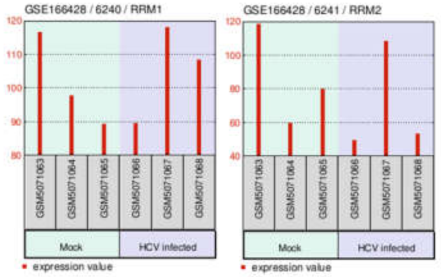

		RRM1		RRM2	
		Value	Average	Value	Average
Mock Huh-7.5 72 hpi	GSM5071063	116.8		118.8	
	GSM5071064	98.07	101.47	59.89	86.37333
	GSM5071065	89.54		80.43	
HCV infected Huh-7.5 72 hpi	GSM5071066	89.8		49.71	
	GSM5071067	118.1	105.4667	108.7	70.72
	GSM5071068	108.5		53.75	

(C)

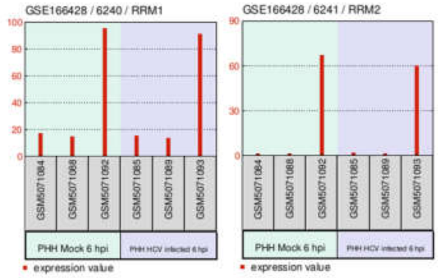

		RRM1		RRM2	
		Value	Average	Value	Average
PHH Mock 6 hpi	GSM5071084	17.69		0.4571	
	GSM5071088	15.17	42.79667	0.3939	22.83033
	GSM5071092	95.53		67.64	
PHH HCV infected 6 hpi	GSM5071085	15.68		0.609	
	GSM5071089	14.27	40.34667	0.4073	20.40877
	GSM5071093	91.09		60.21	

(D)

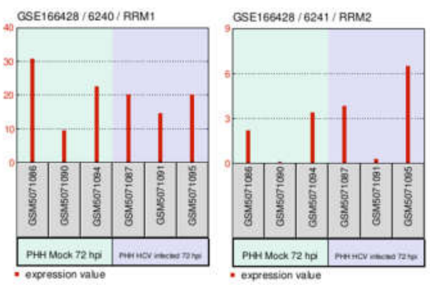

		RRM1		RRM2	
		Value	Average	Value	Average
PHH Mock 72 hpi	GSM5071086	30.93		2.223	
	GSM5071090	9.798	21.186	0.1541	1.948367
	GSM5071094	22.83		3.468	
PHH HCV infected 72 hpi	GSM5071087	20.35		3.869	
	GSM5071091	14.86	18.54	0.3774	3.595467
	GSM5071095	20.41		6.54	

(E)

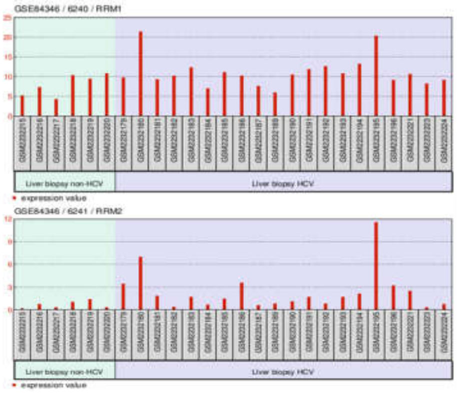

		RRM1		RRM2	
		Value	Average	Value	Average
Liver biopsy non HCV	GSM2232215	5.378		0.2666	
	GSM2232216	7.431		0.8004	
	GSM2232217	4.489	8.037833	0.3916	0.744333
	GSM2232218	10.51		1.156	
	GSM2232219	9.509		1.488	
	GSM2232220	10.91		0.3634	
Liver biopsy HCV	GSM2232179	9.809		3.488	
	GSM2232180	21.52		7.056	
	GSM2232181	9.459		1.946	
	GSM2232182	10.28		0.506	
	GSM2232183	12.47		1.774	
	GSM2232184	7.194		0.7467	
	GSM2232185	11.16		1.575	
	GSM2232186	10.26		3.648	
	GSM2232187	7.698		0.6517	
	GSM2232189	6.123	11.163	0.884	2.441565
	GSM2232190	10.66		1.21	
	GSM2232191	11.95		1.753	
	GSM2232192	12.68		0.9205	
	GSM2232193	10.9		1.793	
	GSM2232194	13.28		2.193	
	GSM2232195	20.35		11.59	
	GSM2232196	9.198		3.31	
	GSM2232221	10.71		2.536	
	GSM2232223	8.299		0.4183	
	GSM2232224	9.26		0.8321	

Supplementary Figure S4. Various RNA sequence data from NCBI GEO database were analyzed. (A) 6 hrs after Huh-7.5 cells were infected with HCV or not (accession GSE166428 at NCBI GEO database); (B) 72 hrs after Huh-7.5 cells were infected with HCV or not (accession GSE166428 at NCBI GEO database); (C) 6 hrs after primary human hepatocytes were infected with HCV or not (accession GSE166428 at NCBI GEO database); (D) 72 hrs after primary human hepatocytes were infected with HCV or not (accession GSE166428 at NCBI GEO database); (E) diagnostic liver biopsies of patients with or without chronic hepatitis C (accession GSE84346 at NCBI GEO database).
